# Correlation between short-time and whole-night obstruction level tests for patients with obstructive sleep apnea

**DOI:** 10.1038/s41598-020-80825-w

**Published:** 2021-01-15

**Authors:** Jeong-Whun Kim, Jae-Cheul Ahn, Young-Seok Choi, Chae-Seo Rhee, Hahn Jin Jung

**Affiliations:** 1 Department of Otorhinolaryngology, Seoul National University College of Medicine, Seoul National University Bundang Hospital, Seongnam, Korea; 2 Department of Otorhinolaryngology-Head and Neck Surgery, CHA Bundang Medical Center, Seongnam, Korea; 3 Department of Otorhinolaryngology-Head and Neck Surgery, Chungbuk National University College of Medicine, Chungbuk National University Hospital, Cheongju, Korea; 4 Department of Otorhinolaryngology-Head and Neck Surgery, Seoul National University College of Medicine, Seoul, Korea; 5 Sensory Organ Research Institute, Seoul National University Medical Research Center, Seoul, Korea

**Keywords:** Diseases, Sleep disorders, Sleep disorders

## Abstract

Identification of obstructive level is crucial for successful surgical outcomes in patients with obstructive sleep apnea (OSA). Unfortunately, most of the dynamic airway evaluations are performed for a short duration under drug-induced sleep; therefore, it is uncertain whether they represent airway events that occur during a whole night of sleep. This study was aimed to evaluate the correlation between obstructive levels that were identified by a short-time and a whole-night test in patients with OSA. Total 101 patients with OSA underwent drug-induced sleep fluoroscopy (DISF) and pressure manometry (PM). For DISF, the obstructive pattern was classified into one of three groups: soft palate, tongue-based, and a combined obstruction. PM was used to measure the proportion of retroglossal events out of total whole-night obstructive events in each patient. The mean age of the patients was 43.8 years. The obstructive pattern was identified as soft palate in 56 patients, combined in 38 patients, and tongue-based in 7 patients following DISF. Results from PM showed that the mean percentage of retroglossal obstructive events was 31.2 ± 30.7%. The average proportion of retroglossal obstructive events that were identified by PM in patients with soft palate, combined, and tongue-based obstruction was 27.2%, 32.1%, and 59.0%, respectively (p = 0.033). There are limitations of evaluating obstructive events that occur during a whole night with short-time tests. Surgeons should be aware the possibility of disagreement in the obstructive level between short-time and whole-night tests.

## Introduction

Obstructive sleep apnea (OSA) is caused by partial or complete obstruction of the upper airway during sleep. When determining surgical approaches, identification of the obstructive level in the upper airway is crucial for successful surgical outcomes^1^. Therefore, various localization methods have been introduced to identify the upper airway obstructive level and structures^2,3^. These methods can be categorized as static or dynamic airway evaluations, short-time or whole-night tests, and drug-induced sleep or natural sleep assessments.

Drug-induced sleep fluoroscopy (DISF)^4–6^ and drug-induced sleep endoscopy (DISE)^7,8^ are commonly performed to examine the dynamic changes of the upper airway during short-time sleep induced by midazolam or propofol. These tests are thought to be superior to other tests, such as nasopharyngoscopy or cephalometry, because in drug-induced dynamic sleep tests, the condition of the upper airway is similar to that during actual sleep. However, these examinations are performed over a short period of time; therefore, the information obtained from these dynamic tests may not represent a full night’s sleep, and surgeons can only characterize the obstructive events occurring during this short time period. Some studies using short-time tests, such as DISE, reported that the obstructive levels changed according to sedation depth^9–11^. The authors have previously reported about pressure manometry (PM) results^12–14^. PM is a whole-night test that is performed with an airway pressure sensing probe inside the upper airway and provides information on full-night dynamic obstructive events during natural sleep. In this regard, this study aimed to evaluate the correlation between short-time and whole-night obstructive level tests.

## Results

### General characteristics of the study patients

A total of 101 patients (90 male patients) were retrospectively reviewed in the study. The mean age, body mass index, and AHI of the patients were 43.8 ± 10.4 years (range, 20–66 years), 26.6 ± 3.4 kg/m^2^ (range, 20.0–35.8 kg/m^2^), and 39.7 ± 24.1 per hour (range, 5.6–102.8 per hour), respectively. The percentages of patients with mild, moderate, and severe OSA were 13.9%, 33.7%, and 52.5%, respectively.

### Obstructive pattern identified by the short-time obstructive level test

Analysis of obstructive patterns by DISF showed that the prevalence of soft palate, combined, and tongue-based obstructive patterns was 55.4%, 37.6% and 6.9%, respectively. There were no significant differences in sex, BMI, and AHI between the three groups (p = 0.782, 0.465, 0.550, respectively); however, there was a significant difference in age between the three groups (soft palate, 44.0 ± 10.5; combined, 45.1 ± 9.8; tongue-based, 34.6 ± 9.9 years; p = 0.046) (Table 1).

**Table 1:** Obstructive pattern with drug-induced sleep fluoroscopy.

	Soft palate (N = 56)	Combined (N = 38)	Tongue base (N = 7)	p-value
Sex (male:female)	51:5	33:5	6:1	0.782
Age	44.0 ± 10.5	45.1 ± 9.8	34.6 ± 9.9	0.046
BMI	27.0 ± 3.2	26.1 ± 3.4	26.6 ± 4.6	0.465
AHI	37.9 ± 21.6	40.9 ± 27.3	47.8 ± 27.2	0.550

*BMI* body mass index, *AHI* apnea–hypopnea index.

### Average proportion of retroglossal obstructive events identified by the full-night obstructive level test

Following evaluation with PM, the average proportion of obstructive events was 31.2 ± 30.7% at the retroglossal site and 68.8 ± 30.7% at the retropalatal site. There were no differences in the proportion of obstructive events according to sex, age, BMI, and AHI (Table 2).

**Table 2:** Proportion of retroglossal obstructive events with pharyngeal manometry.

Characteristics	Number of patients	Proportion of retroglossal obstructive events	p-value
Total	101	31.2 ± 30.7	
**Sex**			0.737
Male	90	31.6 ± 30.9	
Female	11	28.3 ± 30.3	
**Age**			0.775
20–29 years	8	21.9 ± 23.0	
30–39 years	24	36.7 ± 34.9	
40–49 years	44	29.2 ± 29.7	
50–59 years	16	31.3 ± 28.6	
60–69 years	9	34.9 ± 37.1	
**BMI**			0.524
< 25 kg/m^2^	33	28.4 ± 30.9	
≥ 25 kg/m^2^	68	32.6 ± 30.8	
**AHI**			0.862
≥ 5 and < 15	14	30.9 ± 31.0	
≥ 15 and < 30	34	29.0 ± 27.6	
≥ 30	53	32.7 ± 33.0	

*BMI* body mass index, *AHI* apnea–hypopnea index.

### Correlation between short-time and whole-night obstructive level test results

**Figure 1:**
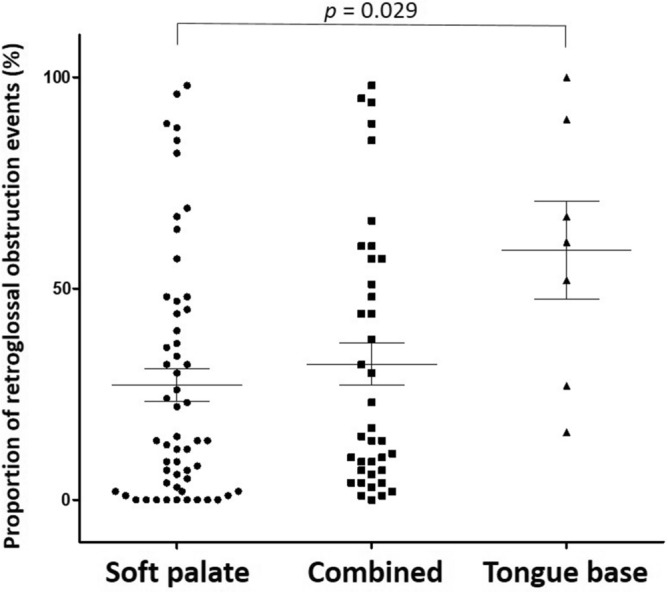
Proportion of retroglossal obstruction according to the obstructive pattern evaluated by DISF. Soft palate (27.2 ± 29.1), combined (32.1 ± 31.1), and tongue-based (59.0 ± 30.6) obstruction patterns.

The proportion of retroglossal obstructive events was significantly different between patients with soft palate (27.2 ± 29.1), combined (32.1 ± 31.1), and tongue-based (59.0 ± 30.6) obstructive patterns (p = 0.033) (Fig. 1).

However, the obstructive patterns that were evaluated by DISF, short-time test, poorly correlated with those that were evaluated by PM, whole-night test. Of the 56 patients who had soft palate obstructive pattern with the short-time test, we observed retroglossal obstructive events in 47 of these patients in the whole-night test, and only 9 patients had 100% retropalatal obstructive events in the whole-night test. Of the 7 patients who had tongue-based pattern, only 1 patient had 100% retroglossal obstructive events in the whole-night test, and 59.0% (range, 16–90%) of the obstructive events were observed at the retroglossal site in the other 6 patients (Fig. 1).

**Figure 2:**
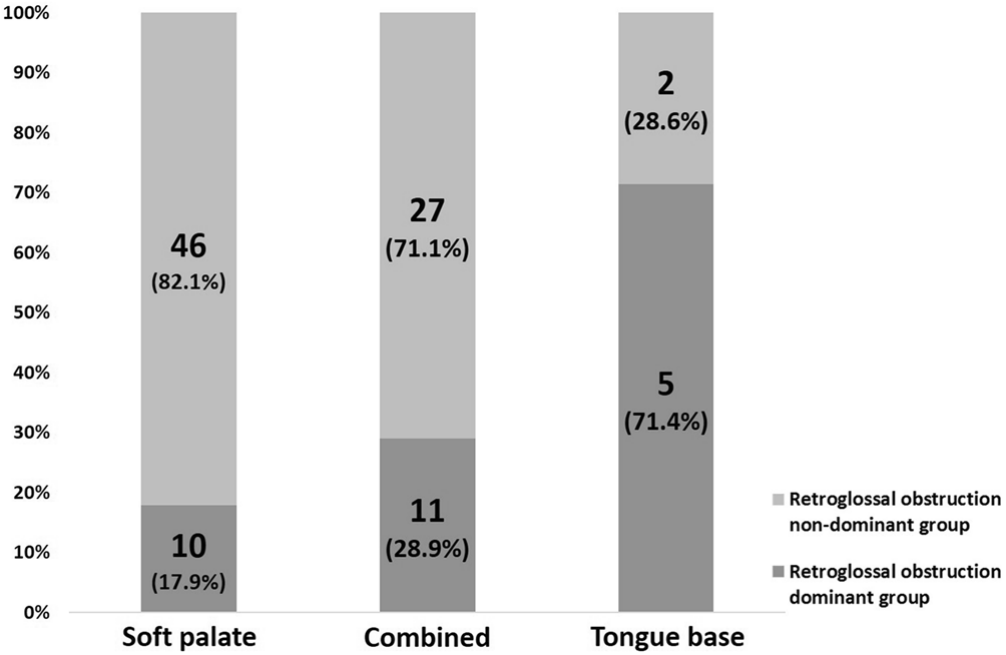
Proportion of retroglossal obstruction dominant and non-dominant groups according to the obstruction pattern evaluated by DISF.

Following evaluation with PM, patients with ≥ 50% retroglossal obstruction were defined as the retroglossal obstruction dominant group and patients with < 50% retroglossal obstruction were defined as the non-dominant group. Following evaluation with DISF, 46 of the 56 (82.1%) patients with soft palate obstruction were defined as the retroglossal obstruction non-dominant group, and 5 of the 7 (71.4%) patients with tongue-based obstruction were defined as the retroglossal obstruction dominant group (Fig. 2).

## Discussion

This study demonstrated that obstructive patterns that were evaluated with DISF, a short-time test, correlated poorly with PM, a whole-night test. These findings may indicate that short-time, induced-sleep obstruction tests do not accurately predict whole-night obstructive events.

The evaluation of obstructive levels is important for sleep surgery, and a variety of methods have been used to identify obstructive patterns in the upper airway of patients with OSA^1–3^. The evaluation of obstructive levels by short-time tests during induced-sleep, such as DISE and DISF, have been commonly used in recent years. DISE uses a flexible nasopharyngoscope to visualize the upper airway under sedation^15^ and classifies obstructive patterns according to the obstructive structure of the velum, oropharynx, tongue base, and epiglottis^16^. DISF is used to identify dynamic obstructive patterns of the upper airway during drug-induced sleep^2,17–20^ and has many advantages. Specifically, DISF is a low cost and quick examination, there is a low risk of radiation exposure, patients are situated in the supine position during evaluation, there is no disruption of sleep during the study, and it allows for the visualization of the whole head and neck, including bony and soft structures and the upper airway^4,5,21,22^. Based on DISF findings, we have established a classification system that assesses the obstructive sites and patterns as soft palate, combined, and tongue-based obstruction^6^.

Classification of obstructive sites by DISF or DISE can have a similar limitation in that findings only represent a short time of drug-induced sleep; therefore, the information that is obtained from these dynamic tests does not represent the events of the whole night, and surgeons can only characterize the obstructive events that occur during a short period of evaluation. Previously, several researchers performed whole-night studies to evaluate upper airway obstruction and reported that apneas may result from obstruction at variable sites in a given patient^23–25^. In addition, because upper airway obstruction is usually aggravated during rapid eye movement sleep, which usually appears one hour after sleep onset^26^, short-time tests are not able to evaluate all of the obstructive patterns that occur during natural sleep.

We have also evaluated obstructive levels during whole nights of natural sleep with PM^12^. PM is a whole-night test that is performed with an airway pressure sensing probe that is situated inside the upper airway and provides information on whole-night obstructive events. PM can be used to assess the proportion of retropalatal or retroglossal obstructive events out of the total obstructive events during natural sleep in a single patient. In our previous study, approximately one-third of the obstructive events in a single patient were associated with retroglossal collapse, and two-thirds were related to retropalatal collapse^12^. This clearly demonstrates the limitations of short-time evaluation by DISF or DISE. In this study, the obstructive patterns that were evaluated by DISF, short-time test correlated poorly with the obstructive events that were evaluated by PM, whole-night test. Fifty-six patients were classified as soft palate obstruction group by DISF, among them 47 patients (83.9%) showed retroglossal obstructive events in PM. Only 9 patients showed 100% of retropalatal obstruction in PM. Among 7 patients classified as tongue base obstruction group in DISF, only 1 patient showed 100% of retroglossal obstruction, other showed 16–90% of retroglossal obstruction. Given that the upper airway obstructive pattern can vary over a whole night in each patient, the simple classification of a single patient as an obstructive pattern on the basis of a short-time examination cannot account for the phenomenon occurring throughout the whole night.

Nevertheless, the role of short-time obstructive level tests for diagnostic examinations cannot be devaluated because DISF complements the limitations of PM. DISF showed that 6.9% and 37.6% of patients had tongue-based and combined obstruction, respectively. When using PM, only the lowest obstructive site can be identified; therefore, retropalatal obstruction cannot be identified even if it were to occur simultaneously with retroglossal obstruction^12^. It is thought that PM and DISF can be used as complementary tests. The authors performed PSG with PM throughout the night and DISF the morning after PSG to evaluate the obstructive levels. These two test results together can be used to determine obstructive levels and surgical extent.

This study has some limitations. First, DISF and PM itself has its own limitations as previously reported^4,6,12,13^. In addition, PM only evaluates whole-night sleep on a specific day, therefore, there is another debatable point that PM also cannot reflect the potential day-to-day variation of the obstructive pattern. Second, with retrospective nature, this study is limited by using DISF as a short time test, not using DISE, which is considered as standard. However, efficacy of DISF for the evaluation of obstructive pattern was also reported previously. Although there are several limitations of DISF, there is a meaning that this study shows some difference compared to the whole-night test. Further study is needed with the DISE results. Third, although body position has been found to have a definite effect on upper airway collapsibility^7,27,28^, this study has a limitation that the effect of body position was not taken into account. This is because PM was measured during natural whole-night sleep and includes all body positions such as supine, right lateral, left lateral and prone, whereas DISF was evaluated only in the supine position during drug-induced sleep. Some of the tongue-based obstructive pattern in DISF could be re-categorized into combined obstructive pattern in case that nonsupine body positions were evaluated. However, given that the number of patients having tongue-based obstructive pattern was small, the results might be similar. A further prospective study is needed in the future to identify the body position effect. Fourth, there are potential bias of DISF classification from the sleep surgeon reviewing the DISF videos. However, interrater reliability of DISF analysis for OSA was excellent in previous report^29^. Finally, we did not use bispectral index monitor to evaluate the level of sedation. Too little sedation may not reproduce the airway obstruction, whereas too much sedation may lead to excessive loss of tone and false positives. Despite those limitations, our study provides novel information about the discrepancy between one of the short-time tests and whole-night tests for patients with OSA.

In conclusion, DISF is a short-time, dynamic obstructive level test, which is less invasive and convenient than whole-night tests. However, there are limitations of evaluating obstructive events that occur during a whole night of natural sleep. Especially, even if there is no tongue-based obstruction in DISF, it does not exclude the possibility of retroglossal obstructive events. Therefore, surgeons should note the possibility of differences in obstructive level findings from short-time sleep studies and whole-night events.

## Methods

### Subjects

We reviewed the medical records of patients who visited the Sleep Center of Seoul National University Bundang Hospital for snoring or sleep apnea and underwent PM during attended in-laboratory full-night polysomnography (PSG) from January 2004 through December 2007. All of the patients underwent DISF to evaluate the obstructive sites of the upper airway on the next morning after PSG. Patients without fully accessible data, with an apnea–hypopnea index (AHI) < 5 per hour, body mass index ≥ 40, or a previous history of palatal surgery were excluded from this study. The detailed protocols in accordance with the Declaration of Helsinki were approved by the institutional review board of Seoul National University Bundang Hospital (IRB No. B-1701/380–103) and the need to obtain informed consent was waived.

### Drug-induced sleep fluoroscopy

DISF was recorded in the supine position on a C-arm table in the fluoroscopic examination room (Allura Xper FD20; Philips, Amsterdam, Netherlands) as previously described^4–6^. Briefly, patients were instructed to lay down in the supine position, and midazolam (0.05 mg/kg) was intravenously administrated to induce sleep. An additional 0.02 mg/kg of midazolam was repeatedly administered to maintain sleep. However, the total dosage of midazolam did not exceed 0.1 mg/kg. Oxygen saturation was monitored throughout the examination. Fluoroscopy generated four 15-s video clips that showed the normo-saturation status before and during sleep and the first and second desaturation events after sleep induction (oxygen saturation dropped by more than 4% as measured by a pulse oximeter). The video clips showed a lateral view of the entire upper airway, including the nasal cavity, oral cavity, pharynx, and larynx and determined the obstructive sites in the upper airway during desaturation events. All video clips were investigated by single expert sleep surgeon who classified the upper airway obstructive pattern into three groups: 1) soft palate, 2) tongue-based, and 3) a combination of soft palate and tongue-based obstruction (i.e., combined obstruction group) as described in a previous study^6^.

### Pressure manometry

The PM, which is a type of catheter manometry with multiple pressure sensors, was composed of a flexible catheter with a 2.7-mm diameter (Gaeltec Ltd., Isle of Skye, Scotland) and four transducers that measured the surrounding pressure. Details of the placement and interpretation of PM have been described previously^12–14^. The catheter was inserted through a nostril and its position in the upper airway was confirmed by lateral radiography. The four pressure sensors on the catheter were located at the mid-esophagus, tip of the epiglottis, tip of the uvula, and nasopharynx. After placement of the PM, patients underwent laboratory level 1 PSG, and the pharyngeal pressures were recorded and presented as additional channels of the PSG as previously described^12–14^. The patterns in the pressure sensors determined the obstructive sites (i.e., retropalatal or retroglossal obstructions) of the upper airway during respiratory distress events. The PM produced sinusoidal waves in all four sensors in patients without airway obstruction; however, flat waves were observed in the nasopharyngeal sensor and sinusoidal waves were observed in the remaining three sensors in patients with retropalatal obstruction. Further, flat waves were observed in the uvular and nasopharyngeal sensors and sinusoidal waves were observed in the epiglottis and mid-esophagus sensors in patients with retroglossal obstruction. The retropalatal and retroglossal obstruction at obstructive respiratory distress events were counted, and the proportion of retroglossal and retropalatal obstructive events out of the total obstructive events was calculated for each patient.

### Full-night polysomnography

The full-night PSG was performed using an Embla N 7000 (Embla; Medcare Reykjavik, Iceland) with standard electrodes and sensors under the supervision of an experienced technician, as previously described^21,22^. The Full-night PSG was used to measure the occurrence of apnea, which was defined as the complete cessation of airflow for at least 10 s; hypopnea, which was defined as a substantial reduction in airflow (> 50%) for at least 10 s or a moderate reduction in airflow for at least 10 s associated with arousals or oxygen desaturation (≥ 4%)^30^; and AHI, which was defined as the total number of apneas and hypopneas per hour of sleep.

### Statistical analysis

Statistical analyses were performed with SPSS version 18.0 for Windows (IBM, Armonk, NY). Results are presented as mean ± standard deviation. Student’s t-tests and one-way analyses of variance with Bonferroni correction with post-hoc analysis were conducted to compare the average proportion of retroglossal obstruction that was evaluated using PM between the obstruction groups (i.e., soft palate, tongue-based, and combined obstruction) that were identified using DISF.

## Data Availability

All the data generated and/or analyzed during the current study are included in this article and are available from the corresponding author on reasonable request.
